# The pandemic experiences of Ontario perinatal providers: a qualitative study

**DOI:** 10.1186/s12913-023-10079-5

**Published:** 2023-10-04

**Authors:** Sigourney Shaw-Churchill, Karen P. Phillips

**Affiliations:** Interdisciplinary School of Health Sciences, Faculty of Health Sciences, University of Ottawa, 25 University Private, Ottawa, ON K1N 6N5 Canada

**Keywords:** Prenatal care, Health care, Pregnancy, COVID-19, Pandemic, Maternity care, Health personnel, Obstetrician, Midwife

## Abstract

**Background:**

The COVID-19 pandemic has produced widespread disruptions for healthcare systems across Canada. Perinatal care in Ontario, Canada was subject to province-wide public health restrictions, reallocation of hospital beds and human health resources. To better understand the impacts of the pandemic on Ontario perinatal care, this study explored the perspectives of perinatal care providers about their clinical COVID-19 pandemic experiences.

**Methods:**

Semi-structured key informant virtual interviews were conducted between August 2021 and January 2022 with 15 Ontario-based perinatal care providers. Recorded interviews were transcribed, and thematic content analysis used to identify major themes and subthemes.

**Results:**

Participants were mainly women, practicing in Eastern and Central Ontario as health providers (obstetricians, nurses, midwives), allied regulated health professionals (social worker, massage therapist), and perinatal support workers (doula, lactation consultant). Major themes and subthemes were identified inductively as follows: (1) Impacts of COVID-19 on providers (psychosocial stress, healthcare system barriers, healthcare system opportunities); (2) Perceived impacts of COVID-19 on pregnant people (psychosocial stress, amplification of existing healthcare barriers, influences on reproductive decision making; minor theme- social and emotional support roles); (3) Vaccine discourse (provider empathy, vaccines and patient family dynamics, minor themes- patient vaccine hesitancy, COVID-19 misinformation); and (4) Virtual pregnancy care (benefits, disadvantages, adaptation of standard care practices).

**Conclusions:**

Perinatal care providers reported significant stress and uncertainty caused by the COVID-19 pandemic and evolving hospital protocols. Providers perceived that their patients were distressed by both the pandemic and related reductions in pregnancy healthcare services including hospital limits to support companion(s). Although virtual pregnancy care impaired patient-provider rapport, most providers believed that the workflow efficiencies and patient convenience of virtual care is beneficial to perinatal healthcare.

**Supplementary Information:**

The online version contains supplementary material available at 10.1186/s12913-023-10079-5.

## Introduction

Perinatal care in Ontario, Canada consists of both regulated health professionals—family physicians, obstetricians (Ob/Gyn), nurses/nurse practitioners, midwives—as well as perinatal support workers such as doulas and lactation consultants [1–3]. Prior to the COVID-19 pandemic, between 2017 and 2019, there were approximately 378,000 births per year in Canada, 38% of these in Ontario [4], with most births occurring in hospitals, supported by medical providers [2, 5]. Canadian perinatal care guidelines emphasize evidence-based, family-focused care that prioritizes women’s autonomy and informed decision making [1, 6] – principles of care intrinsic to midwifery practice [5, 7] and high-quality obstetrical care [8]. A holistic, intersectoral and collaborative approach that is both woman- and patient-centered, enable collaborative decision-making between pregnant people, their families and care providers [6, 7, 9, 10].

In early 2020, about 40% of Canada’s COVID-19 hospitalizations occurred in Ontario, causing extraordinary pressure on the healthcare system, and prompting significant societal changes [11]. The province implemented several community public health measures including mandatory masks, physical distancing, and stay-at-home orders, and multiple region-wide shutdowns lasting two or more months in 2020–21 [12]. Ontario’s hospital capacity was exceeded during the first COVID-19 wave, resulting in suspension of elective surgeries, rearrangements of hospital wards for infection control, and redeployment of patients to other facilities [5, 13]. A provincial task force recognized COVID-19-exacerbation of existing service gaps in the Ontario perinatal healthcare system, particularly for Indigenous, and racialized pregnant people, and those living in remote and rural regions of the province [14]. This task force made several recommendations including the prioritization of perinatal patients for essential medications during supply chain issues, special considerations for rural and remote communities such as access to SARS-CoV-2 tests, and access to universal health care for all, including those not already covered by Ontario’s Ontario Health Insurance Plan (OHIP) [14]. Pregnant patients from Ontario and the rest of Canada reported pandemic-related disruptions to their perinatal health services, particularly the introduction of virtual models of care, and restriction of support person(s) related to provincial and federal pandemic recommendations to limit COVID-19 cases [15–17].

Ontario’s healthcare system, which serves over 15 million people, was significantly impacted by COVID-19, as described [5, 13, 14]. Recognizing that perinatal providers are essential determinants of the quality of patient care, and facilitate patient decision-making [6, 9, 18], we explored the experiences and perspectives of these providers through key informant interviews, to better understand the impacts of the COVID-19 pandemic on perinatal healthcare in Ontario.

## Methods

### Design

This study used a woman-centered care lens [7, 9, 10] to evaluate the impacts of the pandemic on pregnant people’s reproductive decision-making and agency. As essential determinants of woman-centered care, health professionals and support workers in the field of perinatal care were interviewed as key informants in this qualitative study. At the time of recruitment/data collection, the fourth COVID-19 wave, driven by the Delta variant emerged in the Fall 2021, followed by the fifth wave, characterized by the highly infectious Omicron variant in December-Winter 2022 [13, 19]. COVID-19 vaccine uptake (2 doses) reached about 80% of eligible Canadians by December 2021, driven in part by vaccine mandates (e.g. employers-government, healthcare, education, travel) [19]. Public health restrictions in Ontario during this time included vaccine-passports for entertainment and restaurant venues, public mask requirements, and capacity and gathering size limits [19].

### Participants

Key informants were Ontario Ob/Gyn, midwives and perinatal support workers who were (1) English proficient; (2) had worked in prenatal/maternity care field for at least three years; (3) had worked in perinatal/maternity care specifically during the period of January 2020–2022 in the province of Ontario. Candidates who did not meet these criteria were excluded. Key informants, identified through circulation of a recruitment ad via the health sciences researchers’ professional networks, Facebook, Instagram, and snowball sampling, were invited to participate directly through email or Facebook/Instagram messaging, from August 2021 through January 2022. Recruitment continued until thematic saturation was reached.

### Data collection

Building on existing perinatal experiences literature [2, 3, 7] and informed by principles of woman-centered care [7, 9, 10], an interview guide was developed to explore key informants’ COVID-19 experiences and perspectives related to perinatal care, patient decision-making, at-risk communities, and patient-provider rapport (see Supplementary Information). The interview guide included semi-structured questions exploring impacts of the COVID-19 pandemic on (1) participants’ work experiences, (2) perceived patient reproductive decision-making, (3) patient communication- in general, and with minority/marginalized communities, (4) perceived pregnant peoples’ agency in healthcare decisions, including experiences of at-risk patients, and (5) demographics. The interview guide was informally piloted with health sciences graduate students and remained unchanged throughout the formal data collection. Fifteen key informant virtual interviews were conducted from August 2021 to January 2022 by a health sciences graduate student (SS-C), with no prior relationship with participants, until thematic saturation was reached. Online video interviews were captured by audio-recordings and field notes, which were used to produce transcripts.

### Data analysis

Interview transcripts were subject to qualitative data analysis using NVivo™ (Version 1.7.1; QSR International, Lumivero, Denver, Colorado, USA) to generate preliminary codes. Codes emerged inductively and deductively, using the frame of the interview guide, and were then organized into major themes, subthemes and minor themes by thematic content analysis [20]. Major themes represented dominant, recurring concepts expressed throughout the interviews, with subthemes further describing a specific element of a major theme. Minor themes represented divergent concepts expressed by a minority of participants. Preliminary coding was performed individually (SS-C, KPP), followed by integration and identification of themes by team consensus.

### Ethics

The study details, benefits and risks of participation were provided to key informants, who provided informed consent to participate. Permission to conduct this study was obtained from the University of Ottawa Office of Research Ethics and Integrity (REB file number H-05–21-6902).

## Results

### Demographics

Participants included healthcare providers (Ob/Gyn, nurses, midwives), allied health professionals (social worker, massage therapist) – both groups regulated in Ontario – and perinatal care support workers (doulas, lactation consultant). Participants primarily practiced in Eastern Ontario, and the majority identified as women (Table 1).

**Table 1:** Demographics of maternity care providers

Category	Interview participants
Gender	
Male	3
Female	12
Profession	
Allied Health Professional^a^	2
Midwife	2
Nurse	2
Obstetrician^b^	5
Perinatal Support Workers^c^	4
Years of experience	
< 5	2
5 to 9	5
10 to 19	5
20 +	3
Ontario region of practice^d^	
Eastern	10
Greater Toronto Area (GTA)	2
Central	2
Northern	1

^a^Allied health professionals included social worker (1) and massage therapist (1)

^b^Two respondents were Ob/Gyn residents

^c^Major perinatal support workers’ professional roles/titles as follows: doula (3), lactation consultant (1). Two doula participants also identified roles as childbirth/prenatal educators

^d^Cities in each Ontario region as follows: Eastern-Ottawa, Kingston, GTA—Toronto, Central- Kitchener-Waterloo, Northern- Thunder Bay. ON = Ontario

We have presented our findings as major themes with associated subthemes, and divergent minor themes. Four major themes were identified: (1) Impacts of COVID-19 on perinatal care providers*;* (2) Providers’ perceived impacts of COVID-19 on pregnant people; (3) Vaccine discourse, and (4) Virtual pregnancy care*.*

### Theme 1. Impacts of COVID-19 on perinatal providers

The COVID-19 pandemic directly impacted the personal experiences and wellbeing of perinatal providers. Further, pandemic-related changes to the healthcare system created both challenges as well as opportunities for perinatal providers and their interactions with patients. Three subthemes *psychosocial stress; healthcare system barriers; healthcare system opportunities* emerged. *Psychosocial stress* comprised concerns related to providers' fears and concerns about COVID-19 personal exposure and related health impacts, stress related to scientific uncertainties about the pandemic and increased workload (Table 2). Primary health care providers working in hospitals especially experienced work-related stress associated with hospital policies and personal risk of infection. The subtheme *healthcare system barriers* related to providers’ perceptions of changing hospital policies, insufficient human health resources, supply chain shortages and other infrastructure concerns. For some hospital-based providers, the pandemic fostered *healthcare system opportunities*, which enabled rapid implementation of new policies, and fostered new professional challenges and collaboration.

**Table 2:** Major theme: impacts of COVID-19 on perinatal providers

**Subtheme: Psychosocial stress**
*“….I was raising my son, and working and pregnant, and then all of a sudden, the pandemic hit right? Everything shut down March for me and I was due at the end of April. So with that I was forced to go off work about a month and a half earlier than I was going to. I was working like crazy because financially like we couldn't afford for me not to be working. So when I wasn't allowed to work my stress just like through the roof … had my baby in May and I stayed off for six months because that's what made me and my husband felt safe in doing with our family. …. I stayed off longer than I normally would have if there wasn't a pandemic.” (Interview 6, Thunder Bay Registered Massage Therapist)*
*“… I think we still felt the pandemic drain … Some of our nurses were shuffled around a little bit which I know was incredibly draining for them. And then, especially because it was so many unknowns and it seemed like directors were changing….Especially early on when we were just getting a handle on things like a lot of contradictory information that would come out day to day, which is just a bit of a mental drain and I think it was quite stressful and just that added drain coming to work. And then you know the fear for patients as well that they wouldn't always be upfront with us because they would worry that it would impact their care. So if they were having any COVID symptoms they would often lie and say they weren’t because they thought they wouldn’t be seen. And then you walk into a room without full PPE* [personal protective equipment] *and you find a patient coughing away, and you're like, oh thank you for exposing me, if you would have told us we could have just put on our PPE but they didn’t tell us because they were worried we wouldn't see them- which would never have been the case.” (Interview 10, Toronto Ob/Gyn Resident)*
**Subtheme: Healthcare system barriers**
*" I can only speak to my realm as a physician and surgeon, and it has made it exceedingly challenging and exceedingly stressful for the families and also for the care teams. Things take longer, there was a lot of uncertainty in protocol, uncertainty in personal protective equipment-what was required, what wasn't. … we're trying to protect ourselves and others 'cause we don't want to be a vector of infection, but at the same time every second that we take delayed by donning and doffing PPE, scrubbing and scrubbing could result in an adverse outcome. … It makes going to work much more challenging and stressful knowing that everyone is on edge, the demands are high, the resources are low. Whether that's because there's a gap in the supply chain, whether that's because there are people off work because they're quarantining or they're unvaccinated so they’re no longer able to work. With less hands on deck the work still needs to get done." (Interview 5, Toronto Ob/Gyn Resident)*
*“…. we've had to change our whole clinic structure …. so our office wasn’t IPAC compliant* [Infection and Prevention Control (ICAP) Canada]- *meaning that…for infection control… We had to change our whole clinic in terms of* [IPAC]… *we needed new flooring, new couches. All of the nice kind of stuff that made our office feel homey is all gone. Definitely the whole infection control, so just kind of the rigorous cleaning and all of that stuff …. The masking is definitely one thing and then we changed our whole clinic schedule in terms of how often we're seeing people and if some of their appointments are now virtual as opposed to in person.” (Interview 11, Ottawa Indigenous Midwife)*
**Subtheme: Healthcare system opportunities**
*“I actually really love how it* [COVID-19] *opened up everything- to make it even more flexible …. Even for things like family physicians they were never able to bill for phone visits or doing other things and now OHIP* [Ontario Health Insurance Program] *kind of included different codes for them to also be able to be more flexible in how they care for patients which is super awesome. Because you don’t necessarily have to be displaced to have access to good care. If you just have questions, if you just need something faxed somewhere, like if you need a note, like it just more easy to do… And then the flexibility for people to have video appointments … if it’s a time where I don’t necessarily need to see them in person. I really notice people like it. I like it myself” (Interview 1, Ottawa Midwife)*
*“COVID for me was a bit of a godsend… because I made more changes that I had wanted to do in 7 weeks than I did in 7 years… So … we work incredibly hard developing policies and protocols to keep patients safe and keep providers safe. So everything from PPE protocols to categorizing risks in patients … before we had COVID testing to spacing patients out, decreasing the number of visits. A lot of virtual care which I think is wonderful and that would have taken longer to adopt and adapt… And as a leader going through that I probably put in 100 h weeks, but I loved it, because it was a challenge.” (Interview 2, Ottawa Ob/Gyn)*

### Theme 2. Providers’ perceived impacts of COVID-19 on pregnant people

Perinatal care providers openly discussed their perspectives of the effects of COVID-19 on their patients’ pregnancy experiences and challenges. Providers’ perceived impacts of COVID-19 on pregnant people were categorized into three subthemes: *patient psychosocial stress, amplification of existing healthcare barriers,* and *influences on reproductive decision-making* (Table 3)*.* The subtheme *patient psychosocial stress* described providers’ perceptions of patient anxiety and fears around COVID-19 infection coupled with associated isolation, and loneliness. Further, patients expressed to their perinatal providers their sense of loss related to the pandemic-related disruption of their anticipated pregnancy experience. Both perinatal support workers and allied health professionals reported occasions when they assumed *social and emotional support roles* (minor theme) for their distressed patients. *Amplification of existing healthcare barriers* described our perinatal providers’ awareness that their patients’ lacked access to care and support during the COVID-19 pandemic. Existing barriers such as lack of childcare, financial strain, and geographical or travel limitations were perceived to be further magnified in the context of pandemic-related reductions/cancellations in perinatal support services. Finally, providers considered COVID-19 and related hospital policies among the *influences on reproductive decision-making*. Patient distress, anxiety and desire for labor and delivery (L&D) support companion(s) were believed to influence both care decisions and choice of provider.

**Table 3:** Major theme: providers’ perceived impacts of COVID-19 on pregnant people

**Subtheme: Patient psychosocial stress**
*“…what I did see was more things affecting people in the postpartum period…. around week four- week three or week four…I'm really lonely. I feel like I'm losing my mind like and I feel like… I think everybody is dealing with mental health issues right now Thanks COVID. … I feel like with new families that isolation really kicked in.” (Interview 8, Waterloo Doula)*
*“I think COVID has instilled fear. …. And just people are anxious and then postpartum depression is through the roof. Because people can't have, you know everybody over to come and help support them. … afraid of having anybody come over, and afraid of … the baby getting COVID or somebody bringing sickness into the home… and then people who were completely isolated and not having you know, help from their grandma or their auntie or their mom or their friends, so people are sitting at home with a crying, screaming baby, and if your partner has gone back to work? Yeah, it's sad.” (Interview 11, Ottawa Indigenous Midwife)*
**Minor Theme: Social and emotional support roles**
*“…people were feeling alone and grieving what their pregnancy and birth experience and early post-birth experience was going to be. And so we were doing all kinds of free Zoom drop-ins every week where people could just come and share.” (Interview 9, Ottawa Social Worker)*
*“… I think those early ones I had like some due you know March, April, May in those early days and I would spend just hours like crying on Zoom together. It’s loss, like everything was geared up for this type of experience. And to lose that, for those now, who..for those who got pregnant and well I can’t have a doula there.. they kind of didn’t have that expectation but I found in the early part of the pandemic that was the hardest part- it was crushing. It was really crushing for those who were expecting the support.” (Interview 14, Ottawa Doula)*
**Subtheme: Amplification of existing healthcare barriers**
*“A lot* [of northern Indigenous patients]… *come and be admitted for care and then they’d have to quarantine for two weeks before they could go home. And I think it was extremely challenging and extremely isolating. I think there was a few presentations that we saw that were probably later than necessary because patients were hesitant to seek care and resistant to wanting to travel south for care unless it was absolutely necessary. And not even just the isolation, I mean that would suck too, but I'm not, I'm not sure that all of them were… like on paid leave, you know. I’m not sure what kind of financial impact that would have on their ability to care for their families if they were, you know, taken out of work for two weeks on top of however long they needed to be down here.” (Interview 10, Toronto Ob/Gyn Resident)*
*“Transportation can be a barrier for a lot of our clients, and I find one of the biggest barriers right now in the pandemic is definitely for women that have other children at our office. Right now, we are not allowing children into the office. You can now bring one support person with you, but nobody else… Typically, you wanna bring your partner with you, but they might have to stay home to look after the other child. So I definitely think the pandemic and the rules kind of implemented on healthcare facilities right now is a huge barrier to care. And midwifery is really based on… the whole family, and…I really miss having…kids involved and partners involved 'cause it's just not the same, right now.” (Interview 11, Ottawa Indigenous Midwife)*
**Subtheme: Influences on reproductive decision-making**
*“A year and a half in, looking back on it, I think the biggest change and I guess barrier is the lack of having a partner present for visits. In our site the partner is not present for prenatal visits or for ultrasounds, for example. For months they were not allowed to stay in hospital after the baby was born. You know, in the very early days, some of them weren't even present for the births of their own babies…So it's gotten so much better than it used to be and yet I still have patients that are referred to me that choose not to come and see me because they can't have their partner present at their visits….” (Interview 4, Kingston Ob/Gyn)*
*“I think it* [COVID-19]* changed it for a lot of people. Because now what we are seeing… we're seeing a lot of people who are coming in with like crazy high anxiety and you can totally understand and appreciate that… but you know or people coming with really like strict birth plans because they've lost so much control over their lives they try to assert control in another area.” (Interview 13, Ottawa Registered Nurse)*

### Theme 3. COVID-19 vaccine discourse

The introduction of COVID-19 vaccines and related hospital policies created new challenges for providers and their patients, captured as two subthemes: *provider empathy,* and *vaccines and patient family dynamics* (Table 4)*.* The subtheme *provider empathy* described providers’ compassion regarding the challenges presented by COVID-19 vaccines and related decisions faced by their patients, recognizing that hospital policies in particular, were sometimes causes of conflict. The subtheme, *vaccines and patient family dynamics*, described providers’ recognition that within patients’ families, differing views about COVID-19 vaccinations presented significant challenges. Physician-respondents in particular expressed frustration and concern about *patient vaccine hesitancy*, captured as a minor theme. *COVID-19-related misinformation* was identified as a second minor theme limited to Ob/Gyn, nurse and midwifery-respondents. Ob/Gyn in particular attributed vaccine hesitancy to COVID-19 misinformation, with most medical/midwifery respondents recognizing the Internet/social media groups as the leading source of COVID-19 misinformation. COVID-19 myths were noted to cause increased patient fear and anxiety, requiring additional time and resources for medical providers to refute patient misinformation.

**Table 4:** Major theme: COVID-19 vaccine discourse

**Subtheme: Provider empathy**
*“But of all the populations the one where I have a tremendous amount empathy is pregnant individuals. As much as I believe the data would support there is no risk to the fetus …. You know it’s a time when women or pregnant individuals quit smoking, they take care of themselves, they worry about every sniffle. And having a vaccine that, let's be honest, it's very new, it’s a new generation of vaccines… And then there's all the misinformation” (Interview 2 Ottawa Ob/Gyn)*
*“I mean, there's a lot of challenges still around vaccination. … It's sort of this narrative that if somebody is vaccinated that they're safe. And of course, we know that's not true…. so I do have some concerns just around that narrative of this doula is safe and this one is unsafe… Also, concerning, of course, is restrictions around support people and now some restrictions around support people being vaccinated, so I just can't even imagine someone not being able to have their partner there you know at their birth, because of that, because of their partner’s status so that’s disappointing to me.” (Interview 12, Ottawa Doula)*
**Subtheme: Vaccines and patient family dynamics**
*“…I’ve had so many conversations like, ‘oh my mother in law refuses to be vaccinated, but then my mother refuses to come visit me because I still see her because she’s my only source of childcare and I don’t know what to do and it’s causing a huge fight. So the dynamics of inter-family and support and the effect that it has on I think their emotional status in pregnancy ….Folks have really been suffering.” (Interview 1, Ottawa Midwife)*
*“Some families have been really separated by pandemic travel restrictions. So their initial plan for support was family would come, from the States or from overseas or different provinces even. And then they can't get here. So they're alone. Currently, of course with the vaccines. It's an ongoing discussion in all of our [prenatal] classes that families are really struggling with how to set appropriate limits and make decisions around who can come visit or support them if they have a vaccine, don't have a vaccine, if they work in a higher risk environment in the community, or not, and so this additional pressure is pretty high.” (Interview 9, Ottawa Social Worker)*
**Minor theme: Patient vaccine hesitancy**
*“I have a patient… who has COVID. So talking to her about signs and symptoms, and of course she’s not vaccinated… And you don’t want to say I told you so, because I’m sure I talked to her multiple times about getting the vaccine. It's frustrating to see. Like hopefully she’s gonna be fine and most pregnant women who get COVID are going to be fine… but you know, it's frustrating to see this. You know the potential risk that they're putting themselves at when we know that the vaccine is safe, and the vaccine provides protection not just for her, but also it actually provides protection for the baby after delivery …But we are competing against this crazy misinformation.” (Interview 2, Kingston Ob/Gyn)*
*“….I have disappointingly high prevalence of unvaccinated people. I should count to be fair, but at* [clinic] *I think it's about 50:50. So the people that come in and I ask them if they're vaccinated and they say no … The people who are truly vaccine hesitant, at least in pregnancy, I’m not able to shift that position.…. The other thing… our hospital currently does not allow unvaccinated partners to stay in the hospital. So they can come for the birth of the baby but then they have to leave afterwards. And that policy has been in place for a couple months now and I'm still surprised at how little shift there's been in partner vaccination because I would have thought that that would …would be a stimulus… And we have to kind of maintain that professional, compassionate background even, even though sometimes it makes you very frustrated.” (Interview 4, Kingston Ob/Gyn)*
**Minor Theme: COVID-19 related misinformation**
*“I think that's the thing about the Internet is that with enough looking you can find people and information to support whatever your position is. So if you’re a person who is vaccine hesitant you can find a whole community with a whole bunch of information, much of which is misinformation.” (Interview 4, Kingston Ob/Gyn)*
*“People are using- which I commend them for- the Internet, to try to educate themselves. But there's such an epidemic of misinformation that it has clouded certain individuals’ beliefs of what should be done, what’s effective and what’s evidence-based. …you’re having to talk them down, to dispel myths, to use extra time and resources to counteract some of those ill effects of this misinformation. Or perhaps you’ll involve other services because now they may have anxiety or mental health issues that need to be addressed.” (Interview 5, Toronto Ob/Gyn Resident)*

### Theme 4. Virtual pregnancy care

COVID-19 reduced in-person appointment visits, transitioning perinatal care to virtual care. Three subthemes emerged: *disadvantages* and *benefits of virtual pregnancy care,* and *adaption of standard patient care practices* (Table 5). The subtheme *disadvantages of virtual pregnancy care* includes the challenges of provider-patient interactions surrounding pregnancy and COVID-19. Providers described their experiences feeling disconnected from patients and perceptions that information was being missed due to lack of in-person care. *Benefits of virtual pregnancy care* described ease of access for some populations and greater efficiency for routine appointments. *Adaptation of standard care practices* referred to patient care transitions to virtual formats for communication, information sharing, patient tools and consultations.

**Table 5:** Major theme: virtual pregnancy care

**Subtheme: Disadvantages of virtual pregnancy care**
*“I hate virtual. I don't think it's the same as sitting with somebody and being present with them, and being able to touch them if they're telling you something emotional. …You don’t give a client, a hug after they have their baby, which is what we used to always do. So I think virtual has helped us, you know, can keep that access with people. But it’s not the same. And it's limiting, right? Like some people don't have access to a computer or a cell phone … They can't afford that, so it's definitely there's the good and the bad with kind of how everything has had to change in COVID, I feel. But it's definitely impacted the relationship that midwifery tends to build with clients and we don't meet the partners, we don't meet their kids until, maybe we see them in the postpartum, but especially for me, having repeat clients, I want to see their previous kids 'cause I was with them when they had those babies.” (Interview 11, Ottawa Indigenous Midwife)*
*“I think it's a lot easier to build a trusting therapeutic relationship when you are meeting someone face to face rather than on video. And I think it’s just, I don’t know I find video conferences sometimes a little awkward because you don't wanna, conversation just doesn't seem to be as organic like you don't wanna interrupt and I feel like some of the things that patient might have just snuck in and mentioned like off the cuff are not going to come through in video chat in the same way that they would… in a face to-face conversation.” (Interview 10, Toronto Ob/Gyn Resident)*
**Subtheme: Benefits of virtual pregnancy care**
*“Coming to a hospital, waiting in an office, taking half a day off work for the individual, waiting late for a five-minute appointment, it’s inconvenient for patients, it costs money for patients, it's disruptive to their families… I mean I think there's something about the human touch and being in a room with somebody…it’s* [virtual care] *80% as good, but it’s 80% better than being in an office during COVID with someone coughing next to you.” (Interview 2, Ottawa Ob/Gyn)*
*“…Being allowed to communicate with the care provider virtually…. keeping that aspect has been really valuable. Being able to upload videos for example… they can show me stuff … this happened at 3:00 o'clock in the morning and they can show me.” (Interview 7, Kitchener-Waterloo Lactation Consultant/Prenatal Educator)*
**Subtheme: Adaptation of standard care practices**
*“One of the things that we happily decide to do about a year ago is to start to do podcasts, and the first one we did was about COVID and pregnancy… I did another on the vaccine… So, you know, we're basically fighting misinformation on social media with true information. … I think that helped to allay lot of fears, … because we were so proactive in talking to our patients and providing them with the information…* [the pandemic] *really, made people be more creative in terms of how they get the information out there, which I think has been good.” (Interview 3, Kingston Ob/Gyn)*
*“So obviously just not having the capability to be there in person was a huge change. We did do extra prenatal appointments to prepare people…. we would practice out common labor scenarios, but do it over the video call. … we had a whole list of them that were more advocacy and intervention based … …. And we gave them a lot more information than we had before because we weren't there. So one of the things that we do with all of our clients is created labor cheat sheet. So a list of all the different positions and pain management, natural pain management things that we've shown them” Interview 8 Waterloo Doula*

## Discussion

We report here the first published account of the professional and personal impacts of the COVID-19 pandemic on a key informant sample of Ontario healthcare workers and providers, specifically in the context of perinatal care delivery. Our respondents shared their pandemic experiences of evolving hospital policies, emerging and often contradictory science, rapid transitions to virtual care, and increased patient anxiety. Perinatal providers recognized that virtual modes of perinatal care both limited the quality of patient-provider interactions, but also contributed significant efficiencies and benefits, similar to a survey of Western Canadian healthcare providers, which included family physicians and midwives [21]. Perinatal care is essential care, and as such typically exempted healthcare providers and most staff from redeployment to intensive care units during the pandemic [22]. Perinatal care is ideally collaborative, woman-centered care with a foundation of shared decision-making [6, 9]; essential principles challenged by healthcare system adaptations to COVID-19 [22].

### Provider workplace stress, institutional adaptations

Ontario perinatal healthcare professionals and support workers in our sample, like many of their counterparts in other countries [23, 24], experienced workplace-related stress and frustration due to the pandemic. Although increased anxiety and depression were experienced by some New York City perinatal healthcare providers [25], and were reported in a scoping review of studies describing providers’ experiences from multiple countries [26], our respondents conveyed primarily workplace-related stress and concerns about personal infection risk. Workload burden and frustration with the inconsistencies of hospital policies and public health recommendations in the early days of the pandemic contributed to the challenges faced by this cohort of Ontario perinatal providers and support workers – even for those working outside of hospital settings. Implementation of almost universal healthcare policies to limit spread of SARS-CoV-2 such as reductions in prenatal visits, transitions to virtual care, and PPE/barriers to minimize physical interactions between patients and providers [11, 26–28] did not take into consideration the individualized needs of prospective parents seeking pregnancy care [22]. Hospital-based respondents – Ob/Gyn, nurses and to some extent, midwives – seemed resigned to the increased workload, recognizing the necessity of infection control protocols as measures to keep both staff and patients safe. Hospital and public health policies, including cancelled/reduced in-person prenatal appointments, virtual care, and infection control measures, were perceived by some respondents as reducing their capacity to establish connections with their patients beyond their essential clinical roles. It seems evident that pandemic safety measures were barriers to a woman-centered care approach [7–10], such that pregnant patient choice, continuity of care and control were at times sacrificed to comply with hospital and healthcare system policies. Clinical management of COVID-19-infected pregnant patients was not specifically identified as a greater stress for our respondents, in contrast to obstetricians from New York City [25] and the UK [29] who recognized rapidly changing quarantine and PPE policies as factors in their stress and uncertainty when caring for COVID-19-infected patients.

Although providers from multiple countries reported reduced capacity to deliver adequate perinatal care, in part due to limited training, resources and fear of infection [23, 24, 26, 30], this was not described by our participants. In Ontario, hospital infection control policies including PPE, transitions to virtual care, and the restriction of support companion(s) were implemented in early spring 2020 [22], with no apparent increase in adverse outcomes such as preterm birth and stillbirth [31]. Study participants generally did not report concerns about the quality of clinical care provided, however they did acknowledge heightened patient anxiety and distress due to virtual care appointments and policies related to limitations on support companion(s)/visitors – policies which directly contravened the principles of woman-centered care [7–10]. Our sample of perinatal support workers and midwives in particular, were deeply empathetic about their patients’ distress, discussed below, due in part to the restrictive healthcare policies. Further, interviewed doulas described feelings of disappointment about missing their patients’ deliveries, with midwives articulating a sense of loss at their inability to meet patients’ family members, including children they had once delivered. Clear clinical guidelines, updated training protocols, and transparent communication strategies, which include procedures to report concerns, are recommended as strategies to support the adaptation of perinatal care to rapidly changes in healthcare emergencies, including future pandemics [22, 32]. Two of our respondents were pregnant themselves during the pandemic, and although they did not elaborate significantly on their own pregnancies, their experiences are reflective of the substantial occupational risks faced by healthcare providers, as previously reported [33, 34]. Lessons from previous public health emergencies and disaster preparedness policies can be adapted to perinatal care [35–37], but must incorporate the principles of woman-centered care to ensure women’s autonomy and agency are preserved even during disasters.

### Healthcare restrictions to support companion(s)

Experiences of perinatal providers [23, 24, 26–29] and patients [16, 17, 38, 39] from multiple countries suggest that institutional healthcare policies for infection control impaired the quality of woman-centered, perinatal care – a perception echoed by perinatal support workers in our sample. Doulas particularly reported the loss of professional control, as they were often restricted from birth attendance under hospital support companion(s)/visitor limitations. Perinatal support workers also articulated concerns that hospital vaccination policies would further restrict support companion(s) from attending births. For patients, such hospital support companion(s) restrictions were identified globally as major COVID-19 perinatal stressors, with most global healthcare settings banning partners from prenatal appointments and limiting birth attendants to the period of active labor and immediately postpartum [30, 38, 39]. Support companions, recognized as essential components of woman-centered care [9, 10], improve pregnancy outcomes, facilitate rapport with healthcare providers and can enhance agency during reproductive decision-making [40, 41]. Support companions may be partners or perinatal support workers, such as the doulas interviewed in our study. Although traditionally described as labor or birth companions [40, 41], we recognize the contributions of support companion(s) throughout the perinatal period, including during prenatal appointments. Our perinatal health professionals described their patients scrambling to include partners on the phone or video-appointments, consistent with experiences from the UK [42, 43] and Australia [44], demonstrating respondents' perceived impacts to patients’ reproductive decision-making and support. Restrictive support companion(s) hospital policies were perceived by perinatal support workers in our study as distressing to their patients, who increasingly sought reassurance and support from their providers. Although perinatal healthcare providers commonly serve as educators and medical system guides in addition to their clinical roles [18], some of our respondents felt an increased psychological burden as they served as emotional surrogates in the absence of partners. Certainly it is now evident that hospital policies restricting support companion(s) during both prenatal care and L&D exacerbated patients’ experiences of isolation and loneliness, and contributed to adverse perinatal mental health [16, 30, 38].

### Virtual patient care- benefits, disadvantages and adaptations

For decades, virtual healthcare or telehealth has complimented in-person care and has gained acceptance as a strategy to improve healthcare access for remote and rural patients due to the geographic realities in Canada [21, 45, 46]. Unlike most Canadians, Indigenous women in remote regions of Canada must often travel great distances to receive perinatal care as their communities lack hospitals, obstetricians, or other perinatal care providers; factors contributing to poor perinatal health outcomes [47]. Our sample of Ontario perinatal health professionals acknowledged that the abrupt transition to widespread virtual perinatal care modalities required adaptations to new technologies, and adjustments to novel patient interactions. In general, respondents acknowledged many benefits associated with virtual care, including reduced COVID-19 exposure, improved accessibility for some patient populations, and improvements to their own workflow, consistent with rural Western Canadian healthcare providers [21] and a global survey of perinatal providers [48]. Similarly, scoping and systematic reviews [26, 27, 49] report that providers perceive virtual care as a generally acceptable form of delivering perinatal services with the capacity to improve access to care. Respondents appreciated virtual care’s reduction of transportation, childcare and time constraint barriers to perinatal care access, particular for their rural and remote patients. They also recognized that some patients would face challenges to virtual care due to limited high speed Internet access and low digital literacy, consistent with previous studies [21, 48]. Participants were concerned that COVID-19 further exacerbated already limited postnatal services in rural and remote communities.

Despite the efficiencies and healthcare access improvements for some patients, ultimately our participants asserted that virtual care is not a substitute for in-person care. Respondents agreed that virtual care reduced their perceptions of personal connection and relationship-building opportunities with their patients. The relationship between perinatal care providers and their patients is an essential aspect of woman-centered care, with the establishment of trust-based relationships identified as a key factor in patient satisfaction and their likelihood to seek future perinatal care [6, 9, 10, 18, 50]. These findings suggest that our sample of Ontario perinatal care providers sometimes struggled to develop this relationship through virtual care, highlighting the importance of ensuring patient-provider rapport even through pandemic-induced healthcare system changes. Further, participants discussed the importance of being able to see their patients during virtual consultations and were concerned about missed cues from body language, not being able to physically examine patients or provide hands-on emotional support, which they described as a standard part of their clinical care. Our findings are consistent with the global experiences of perinatal health professionals, who framed the use of patient masking, physical distancing, and virtual patient care as impersonal and dehumanizing, associating these practices with reduced relationship building capacity [23, 24, 26, 30, 48]. Canadian perinatal mental health providers recognized the transition to virtual care as worsening patients’ isolation, anxiety and created privacy concerns for virtual consultations [51]. Integration of virtual visits within perinatal care models will undoubtedly remain a regular feature of healthcare for many settings given the high level of provider satisfaction ratings across studies [48, 49]. Optimization of virtual perinatal care must address inequities related to diminished accessibility for individuals with disabilities, socioeconomic or technical Internet service challenges [45, 48], and ensure that virtual care complements but does not replace in-person clinical care.

### Providers’ perceptions of their patients

As described, Ontario perinatal care providers in our study were sensitive to the pandemic and healthcare policy-impacts on their patients’ experiences of pregnancy and delivery. Given the provincial stay-at-home orders [13] and resulting self-isolation practiced by many pregnant patients for fear of COVID-19 exposure, interactions with health professionals in our sample often served as opportunities for surrogate socialization. During these perinatal visits, providers learned of their patients’ increased stress and anxiety, complicated by their lack of social support and social isolation. Even prior to the COVID-19 pandemic, poor social support during pregnancy was an established risk for depression, anxiety, and self-harm [51, 52]. Perinatal mental health services, like most healthcare, was impacted by the pandemic [38, 51], exacerbating pregnant patients’ experiences of psychological distress and mental illness [16, 51, 53, 54]. The intersections of patient characteristics such as language barriers, recent immigration, forced relocation for perinatal services, Indigenous and racialized identities, and disability contribute to limited social support and adverse perinatal mental health [47, 51]. Systemic racism and the legacy of colonialism are established healthcare barriers in Canada [55, 56], the US [57] and many countries [23, 58, 59], which contribute to poor pregnancy outcomes despite Canada’s ‘universal’ healthcare system [60, 61]. Despite the efficiencies of virtual care, our respondents perceived that structural barriers to perinatal care access were amplified during the pandemic, consistent with previous studies [17, 30, 58, 59]. Participants acknowledged that their patients with high levels of anxiety, competing childcare demands, low socioeconomic backgrounds, immigrants, racialized or Indigenous were particularly at risk during the pandemic, reflecting exacerbation of societal and health disparities during disasters like pandemics [34].

Although pregnancy was identified as a priority condition for COVID-19 vaccination by April 2021 in Ontario, COVID-19 vaccine uptake among pregnant people was substantially lower (71.2%) than reproductively-aged women in the general Ontario population (88%) [62]. Ontario medical providers interviewed here, expressed both frustration and concern about patients’ vaccine hesitancy, believed to be fostered by social media-based misinformation. Time spent addressing patient vaccine hesitancy increased workloads and was yet another source of provider stress during the pandemic. Although participants were empathetic to the genuine concerns expressed by their patients about the potential teratogenic risks of COVID-19 vaccines and long-term health outcomes for their babies, medical perinatal providers in particular, described considerable efforts to encourage COVID-19 vaccination and combat misinformation. The launch of COVID-19 vaccines was initially heralded with hope for a speedy end to the pandemic, however this optimism quickly eroded as variants emerged, community restrictions and vaccination mandates were introduced, and public confidence began to wane [19]. Social media-fostered misinformation promulgated myths that COVID-19 vaccination caused adverse reproductive affects including infertility, miscarriage, and stillbirth, which contributed to vaccine hesitancy [63]. There is a substantial literature recognizing the pivotal role of the perinatal provider as an essential determinant of vaccine uptake (influenza, Tdap- tetanus, diphtheria, and pertussis) during pregnancy [63–65]. Ultimately, the quality of the provider-patient relationship in terms of respect, trust and transparency along with the provider’s willingness to continue to offer vaccines even after initial patient refusals, may mitigate vaccine hesitancy during pregnancy [63, 64]. Even post-pandemic, misinformation driven by social media will continue to challenge evidence-based perinatal practice, with suggestions that a woman-centered care approach requires development of practitioner skills in ‘infodemic management’ to address patient perinatal health misinformation successfully [66].

## Limitations

The qualitative design of this study is a strength, which enabled participants to contribute their perspectives and experiences of perinatal care in Ontario. This study also provides a uniquely heterogeneous sample, such that the diversity of providers highlights the many different aspects of perinatal care that were influenced and changed by the pandemic. There were, however, some limitations to this work which should be considered. First, this qualitative study is not generalizable to the Ontario perinatal provider population and is not meant to be representative. Second, despite efforts by the research team to recruit a geographically diverse sample, most of the sample were from Eastern Ontario, such that rural and remote maternity care providers, as well additional respondents serving large, diverse urban populations, would have contributed valuable perspectives of their experiences during the pandemic. Finally, as this study is focused on provider perspectives, additional research is required to gain patient perspectives of their pregnancy care experiences.

## Conclusion

Ontario perinatal care providers described workplace stress and increased workloads due to the COVID-19 pandemic, vaccine hesitancy and general misinformation. Providers perceived that their patients were distressed both by the pandemic, but also by hospital policies which limited support companion(s), in-person appointments and reduced perinatal care services. Amplification of existing social, economic, and other patient barriers to care was attributed to the pandemic by providers. Finally, although virtual care reduced capacity to provide interactive patient care, providers generally accepted the efficiencies and accessibility of virtual care as an appropriate complement to in-person perinatal care.

### Supplementary Information

**Additional file 1.**

## Data Availability

The data (interview transcripts) that support the findings of this study are available from the corresponding author (KPP) upon reasonable request, but restrictions apply to the availability of these data, and so are not publicly available. Only publishing/dissemination of quotations was approved by University of Ottawa Research Ethics Board, not the entirety of original interview transcripts. Similarly, study consent forms to participate in interviews only authorized researchers to use selected quotations not entire transcripts for publication/dissemination. Selected quotations appear throughout the article, reproduced with permission.
