# Supplementary material for: The pandemic experiences of Ontario perinatal providers: a qualitative study
Source: BMC Health Serv Res. 2023 Oct 4;23:1057. doi: 10.1186/s12913-023-10079-5 (PMC10552313; doi:10.1186/s12913-023-10079-5)
Supplement: Supplementary file 1 — Additional file 1. [file 12913_2023_10079_MOESM1_ESM.docx]

Supplementary Information

Interview Guide

1. In what major ways has your work been impacted by the COVID-19 pandemic?
2. In what ways has COVID-19 changed reproductive decision-making for your patients/clients?
3. Do you have any concerns about how COVID has impacted the ways in which you communicate with your patients in general?
4. Do you have any concerns about how COVID has impacted the ways in which you communicate with your patients from minority or marginalized groups?
5. Do you perceive that COVID has reduced pregnant people’s agency in general regarding healthcare decisions?
   1. Has COVID reduced the agency of pregnant people from at-risk communities?
6. Is there anything else you would like to add about your experience caring for pregnant individuals during the pandemic?
